# Randomly overlap subarray feeding network to reduce number of phase shifter in 28GHz

**DOI:** 10.1371/journal.pone.0277404

**Published:** 2022-12-08

**Authors:** Maryam Shadi, Zahra Atlasbaf

**Affiliations:** Department of Electrical and Computer Engineering, Tarbiat Modares University, Tehran, Iran; Edinburgh Napier University, UNITED KINGDOM

## Abstract

Synthesizing antenna arrays for fifth-generation communication technology is the most significant issue in the electromagnetic industry and academia. This paper focused on a comprehensive algorithm for developing a 5G base station antenna array. The suggested algorithm aims to provide a high-gain array antenna with a continuous wide scan angle without a grating lobe, as much as a compact size, low cost, and simplicity of fabrication, especially in the array feeding network system. The best architecture is specified by comparing the array factor of numerous subarray combinations to achieve the grating lobe’s minimum level. By considering additional limitations in our approach, such as different subarray symmetric architecture, complex weighting function, minimal number of overlapped elements, and an optimal number of microstrip layers, we improve the specification over previous research and lower the runtime procedure. The proposed method is also used to construct a linear array antenna with 49 radiating elements for a 5G base station antenna operating at 28 GHz. Consequently, the number of phase shifters has been reduced by more than 53%, significantly improving over earlier efforts. Then a hybrid genetic algorithm and a particle swarm optimization technique are applied to determine the optimal values of excitation coefficients to control side lobe level(SLL) and beam scanning. The amplitude and phase step variations are calculated as 0.1 and 1°, respectively. HPBW of 2.8°, gain of 28 dB, scanning up to ± 25° in one direction, and SLL below -24 dB are the electromagnetic properties of the designed aperiodic linear array. An example of implementing the suggested method, a 16-element array with a random overlap subarray structure, including the feeding network and microstrip antenna element, will be modeled using a full-wave simulator. The simulation results show that the proposed algorithm is efficient for designing array topology.

## Introduction

Wireless communication technologies have grown significantly during the past decade, and various generations of communication networks have been proposed. Fifth-generation (5G) cellular network technology has higher data rates with more energy efficiency and lower latencies [1]. Base station (BS) antennas play a crucial role in 5G communication, so a comprehensive design of antenna arrays for 5G systems is required [2]. Preliminary research on the upcoming generation’s technologies increasingly recommends utilizing the millimeter-wave spectrum [3]. Therefore, the frequency allocated to the 5G communications is more than 24GHz [4]. The BS antenna should have a high gain to cope with the losses in the millimeter-wave band. Moreover, to prevent wave propagation in all directions is essential to narrow beamwidth with a wide scanning angle without grating lobes for 3D space coverage [5]. Different factors such as scanning resolution, scanning speed, variation of HPBW and gain, and lateral lobe level influence its reliability. In other words, beamforming (BF) is another technology critical for the fifth generation’s communication. There are several methods to BF, including the mechanical process, phased array antennas [6] (analog BF, digital BF, and hybrid BF [7]), metamaterial antennas [8], reflector antennas, traveling wave antenna [9], and switched beam antenna [10]. A phased array antenna with hybrid, digital, and analog BF is a promising solution for multi-user (MU) or single-user (SU) massive MIMO [11]. MU-massive MIMO system has a large number of antenna elements. Various synthesis methods have been presented in the literature to design phased array antennas based on the mentioned conditions, categorized into optimization-based methods, theoretical methods, and numerical methods. In the first category, a genetic algorithm (GA) [6, 12], particle swarm optimization (PSO) [13, 14] and hybrid of them as GAPSO(genetic algorithm and particle swarm optimization hybrid) [15] are the most prominent algorithm to determine the amplitude, phase, and space tapering [16]. In the theoretical solution group, sinusoidal, Bessel, and Legendre polynomials [17–19] are used to determine the best location of the elements for designing a non-periodic array in different coordinates. Numerical methods such as non-uniform fast Fourier transform also are used for synthesis array antenna [20, 21]. Regardless of the synthesizing techniques, the phased array antenna has many antenna elements. Consequently, they need a large number of RF devices such as phase shifter (PS), variable gain amplitude (VGA), filter, and mixer. Indeed, high-speed digital signal processing is required, as well. The number of PS is more critical in the phased array antenna. Even though there is much improvement in phase shifter fabrication, large-scale phase shifters are not affordable [6, 22]. A uniform subarray (USA) approach was introduced several years ago to reduce the number of phase shifters [23]. Thus, the USA is composed of two arrays, the primary array elements are antenna, and the secondary array elements combine the primary arrays (subarrays). Although the RF device’s number is reduced because of too large space (concerning the wavelength) between the subarrays, the grating lobe (GL) has occurred. Furthermore, the wide scan angle is not achievable utilizing the USA technique. Two methods are proposed to address the GL problem in phased array antennas. The first method decreases the distance between the secondary array elements by the uniform overlap subarray (UOSA) technique [20–25]. The second technique uses an aperiodic array that is achieved by random subarray (RSA) [10, 18], and randomly overlap subarray (ROSA) [20–24, 26]. In some of these cases, in UOSA, the feeding network is more complicated, and an aperiodic structure may have complex construction. Besides, the designs have been done in previous works without providing conditions and limitations to choosing the topology type for antenna arrays. Moreover, their algorithm needs more runtime. So, there is no comprehensive approach to designing a cost-effective antenna in cases with different values of electromagnetic parameters. GeneralizedIn this paper, an executive yet effective algorithm is proposed to synthesize the array antenna based on desired specifications that consist of specific values of gain, HPBW, RF band, SLL, and scan angle. Some limitations optimize the runtime of our algorithm, and the desired result is determined earlier. Also, these limitations reduced complex of construction [27].

Amplitude, phase, and space tapering can be determined according to the trade-off between minimum cost, simplicity of construction, and technical specification. Amplitude and phase tapering is highly dependent on cost with different choice. Amplitude tapering can be used on both the primary and secondary arrays, or just one of them, while phase tapering can be used on the secondary array [10, 28–32].

The methodology design of the aperiodic array based on the GAPSO algorithm will be described in section II. Section III shows the proposed approach for developing a phased array antenna and the simulation result of the constructed linear array antenna for 5G communications. This section also includes the results of a simulation of the proposed array’s feeding network with an antenna element. Then, the most recent methods are selected, and their performance is compared with the proposed method. Finally, in part IV, the work’s conclusions are presented.

## General method to design array antenna

The affordable phased array antenna architecture is based on the technique of subarraying. In this context, the number of elements is assumed to be the first group to be recognized as the primary array (shown with *N*_*p*_), and the array whose elements are the first group is considered the second one (offered with *N*_*psh*_). The far-field pattern for a linear subarray of isotropic elements is assumed in the following equation.

$$\begin{array}{c} AF\left(\theta \right)=\sum_{p=1}^{N_{psh}}b_{p}\sum_{q=1}^{Np}a_{pq}e^{\left(j\frac{2\pi }{\lambda }d_{i}\;\text{sin}\;\theta \right)} \end{array}$$ (1)

The above parameters are defined in Table 1.

**Table 1:** The definition of parameters of Eq 1.

Heading1 Heading2	
*b*_*p*_	Complex weight in subarray p
*N*_*pSh*_	Subarrays number
*a*_*pq*_	Amplitude weight at subarray p of element q
*N*_*p*_	Number of subarray’s elements
λ	Wavelength
*d*_*i*_	The distance of element i to wavelengths from the center of the array.*i* = (*p* − 1) * *N*_*x*_ + *q*

$N_{x}^{\prime }s$ value controlling the distance between the elements depends on a type of array topology according to Eq 2.

$$\begin{array}{c} N_{x}=\{\begin{array}{cc} N, & USA\\ N/2, & UOSA\\ (R_{m-1}+R_{m})/2, & RSA\\ \left(N_{m-1}+N_{m}\right)/2-RO_{m}, & ROSA \end{array} \end{array}$$ (2)

**Fig 1:**
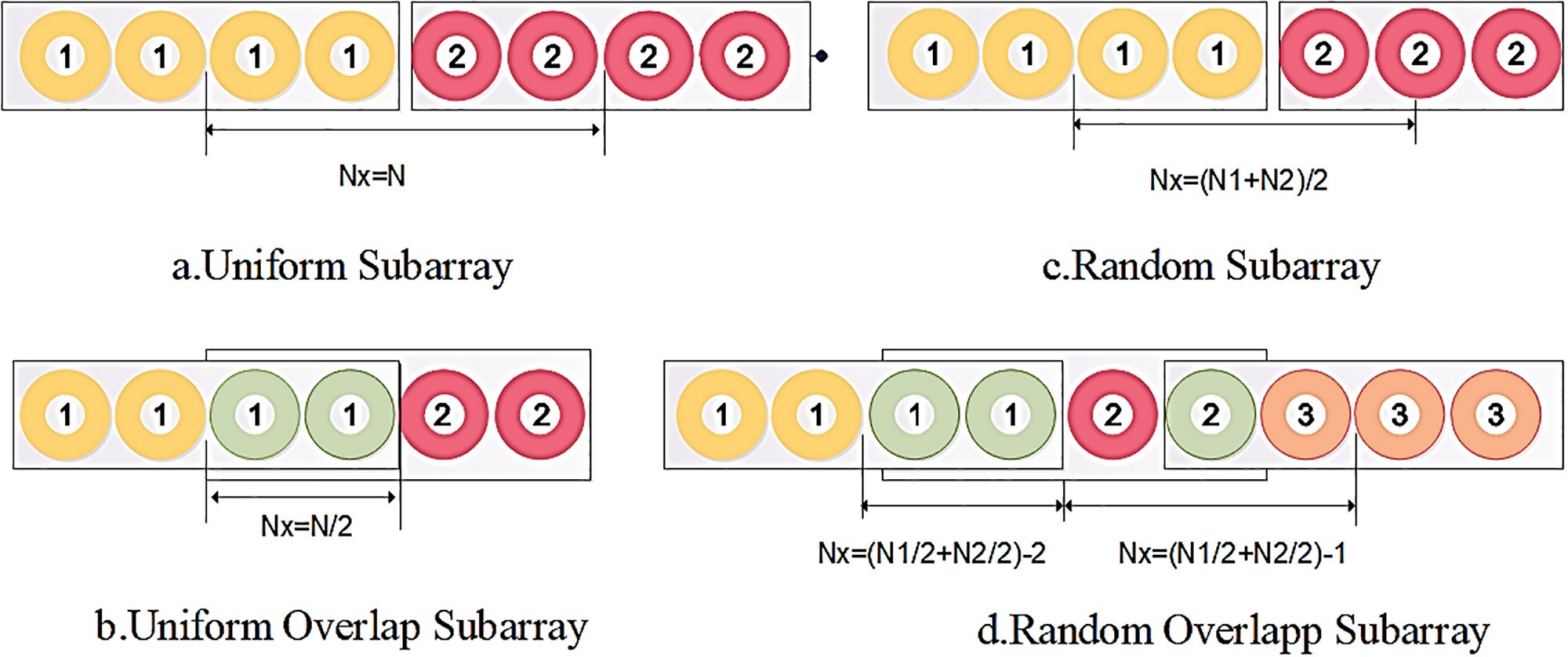
Show parameter *N*_*x*_ for different topologies. a. Uniform Subarray, b. Uniform Overlap Subarray, c. Random Subarray and d. Random Overlap Subarray.

*R*_*m*_ is the number of subarray components in the random array, *RO*_*m*_ is the number of subarray components used in the next subarray in ROSA topology, and m shows subarray number. Fig 1 shows the above relation to four different topologies. Yellow or red color represents each subarray, and for delivering the overlap, elements used the green color. Other values of *N*_*x*_ construct an aperiodic array to have the best topology. Three parameters, *b*_*p*_, *a*_*pq*_, *d*_*i*_ are essential to have the desired radiation pattern. We choose another variable and rewrite the Eq 2 to explain the concept of the method, assume the Eq 3:

$$\begin{array}{c} \begin{array}{cc} AF(u) & =\sum_{p=1}^{N_{psh}}b_{p}\sum_{q=1}^{Np}a_{pq}e^{\left(j\frac{2\pi }{\lambda }d_{i}u\right)}\\ u & =\text{sin}\;\theta \;0\leq u\leq 1 \end{array} \end{array}$$ (3)

To have more comfortable fabrication, the array elements are symmetrical, so we consider the synthesis problem in the spectrum 0≤ u≤ 1, or 0≤*θ*≤ *π*/2 radians. The equation is converted to discrete form to determine the unknown parameter in Eq 4 by considering J points in variation angle (*θ*).

This equation is sampled in J points shown in Eq 4.

$$\begin{array}{c} \begin{array}{cc} AF(u_{j}) & =\sum_{p=1}^{N_{psh}}b_{p}\sum_{q=1}^{Np}a_{pq}e^{\left(jk\beta_{q}\right)}.\;j=0,1,‥J-1\\ k\beta_{q} & =\frac{2\pi }{\lambda }d_{i}\;,u_{j}=\frac{2\pi }{\lambda }d_{i}\delta \;,u=\frac{2\pi }{\lambda }d_{i}j\frac{1}{j-1} \end{array} \end{array}$$ (4)

For more comfortable fabrication, the array elements are symmetrical, so we consider the synthesis problem in the spectrum 0≤ u≤ 1, or 0≤*θ*≤ *π*/2 radians. The equation is converted to discrete form to determine the unknown parameter in Eq 4 by considering J points in variation angle (*θ*).

As a result, a multidimensional non-linear system with an amplitude vector, desired pattern vector, and a non-linear function of the distance between elements in each point is formed, as described in Eq 5.

$$\begin{array}{ccc} \begin{array}{cc} & \left[\begin{array}{c} AF(u_{1})\\ AF(u_{1})\\ \ldots \\ AF(u_{1}) \end{array}\right] \end{array}= & \left[\begin{array}{cccc} X(u_{1},d_{1}) & X(u_{1},d_{2}) & \ldots & X(u_{1},d_{i})\\ X(u_{2},d_{1}) & X(u_{2},d_{2}) & \ldots & X(u_{2},d_{i})\\ \ldots & \ldots & \ldots & \ldots \\ X(u_{j},d_{1}) & X(u_{j},d_{2}) & \ldots & X(u_{j},d_{i}) \end{array}\right] & \left[\begin{array}{c} I1\\ I2\\ \ldots \\ Ii \end{array}\right] \end{array}$$ (5)

Solving this non-linear system manifests the distance between elements in the array by assuming the amplitude and vector of the desired pattern are already known. As a result, a distance between the subarrays might determine the number of phase shifters, an essential parameter in the phased array antenna. Because there is no analytical solution for this non-linear system, we must concentrate on numerical and optimization procedures, which will be discussed in the next section.

### 0.1 Guideline design

**Fig 2:**
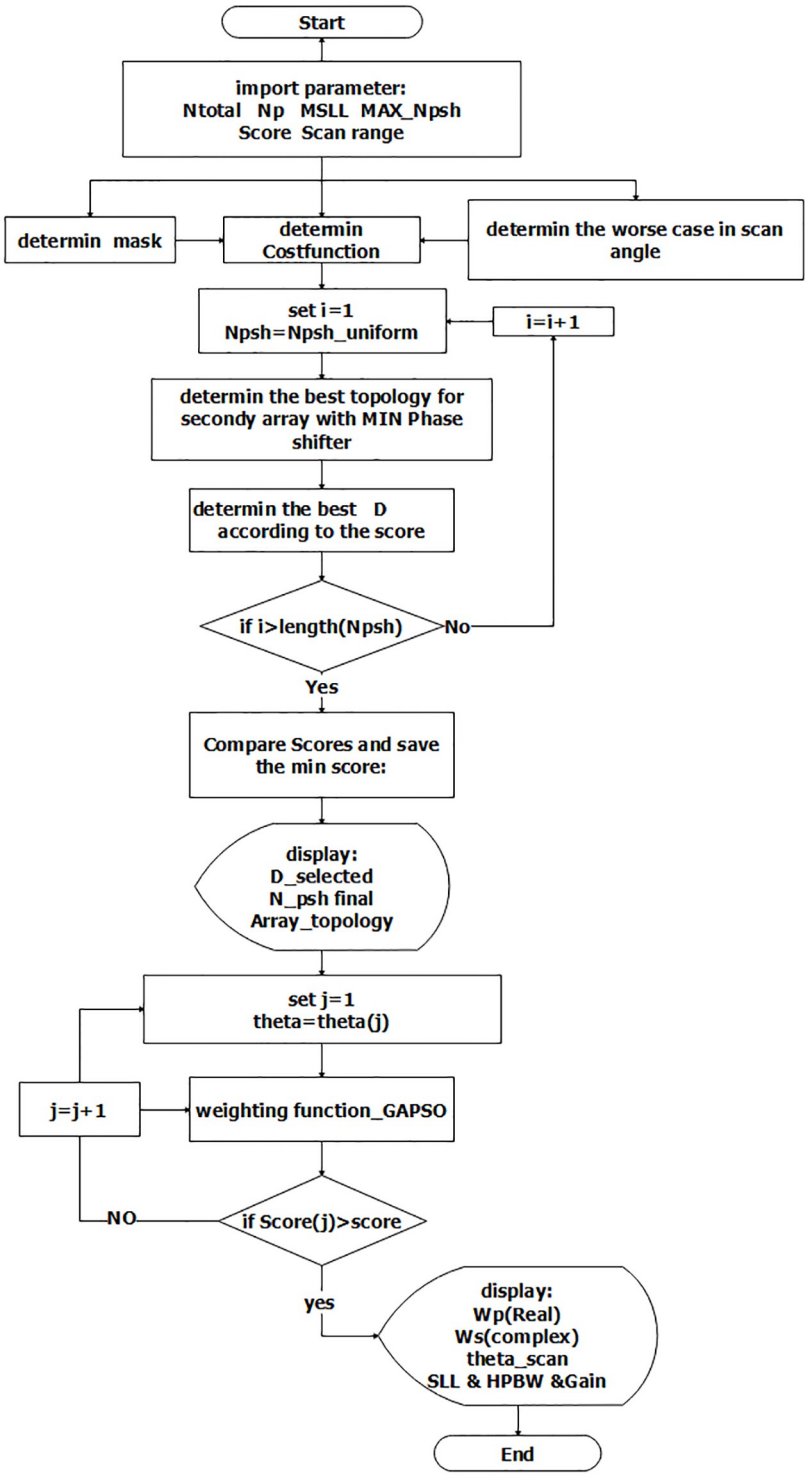
Guideline of the proposed methodology.

Any linear phased array antenna can be synthesized using the illustrated MATLAB algorithm. The flowchart of the designed algorithm is demonstrated in Fig 2. In the proposed algorithm, the comprehensive solution is considered. In other words, the desired topology of the array can be chosen from a periodic array such as a conventional array (without using subarray technique), USA (uniform subarray), UOSA (uniform overlap subarray), and aperiodic arrays such as RSA (random subarray)and ROSA (random overlap subarray). At first, gain, HPBW, scan angle, maximum SLL, the maximum number of the phase shifter, and the complexity of the feeding network should be defined. Then, the preliminary data could determine the total number of elements (*N*_*t*_) and the number of primary array Elements (*N*_*p*_). The USA will be determined by having the value of *N*_*t*_ and *N*_*p*_. It is noticeable that this topology (USA) uses the least number of phase shifters among all typologies. So we can be considered as a reference in our algorithm. Generally, the desired topology is chosen by comparing the radiation patterns of various structures to a predetermined cost function. Based on the USA as a reference, the worst angle condition (the angle with GL or high level of SLL) in the scanning range is estimated. We determine the proper topology according to the angle to ensure better specifications can be obtained instead of using the boresight angle to design. Although the variation of the gain and HPBW in the scanning angle is not ideal, we consider that these parameters should not change as much as the reference array does in the USA.

Now, it is time to determine the distance between the subarrays; in other words, what the vector d in Eq 6 does. To determine vector d, we have two approaches: Firstly, we consider a similar uniform primary array (*N*_*p*_ element per subarray) with similarity in excitation coefficients to have simple fabrication. We assume a different condition relating to the repeatable use of an element to generate each subarray to define the type of topology. We will have the USA if each component is only used in one subarray, and overlapping will occur if it is used in more than one subarray. We have UOSA when they are used only twice and ROSA when they are utilized equal or more than twice at random. Secondly, we choose a different number of elements for primary array *N*_*p*1_, *N*_*p*2_, and others, so we have RSA. Just the symmetrical arrays are used in this approach. According to the *N*_*t*_, *N*_*p*_, and the phase shifter’s minimum number, all combinations of the second array are matrix. This matrix shows different subarray placements next to each other. According to previous work, the dimension of this matrix is massive. We assume three conditions at once to reduce the matrix dimensions: the array is symmetrical, its length is constant, and no element is used more than four times. As a result, some of the combinations have been omitted. The array factor of the residual combination by the minimum number of phase shifters is calculated. Based on the cost function and the value of the SLL, the related score is determined. Then, the number of phase shifters is increased and repeated the same procedure for each phase shifter up to a maximum number of phase shifters. The final topology array could be chosen by comparing the value of each array’s score related to each phase shifter. The amplitudes of primary arrays and amplitudes and phases of the second array are determined simultaneously using the GAPSO optimization algorithm, to form a pattern. The main beam for assessing the optimum scanning angle is steered in small-angle increments (typically 0.1°) until the level of the SLL reaches the maximum allowable side lobe level (MASLL). This scan angle is reported as the actual maximum angle of the scan. As a result, the maximum scanning angle and SLL are inversely proportional, with a trade-off between them.

We used the hybrid algorithm to get better results and to achieve the advantages of both genetic algorithm (GA) and particle swarm optimization (PSO) simultaneously [33, 34]. Thus, the first half of the population is optimized with the GA, and the remaining half will join the PSO algorithm. The optimization process will continue until the end conditions are met. As aforementioned, the array factor is affected by the distance between elements, excitation coefficients, and antenna scan angle. So, an explanation of the minimax criterion follows:

$$\underset{\theta }{\text{max}}\;\underset{N_{psh}}{\text{min}}[\|AF(d_{n},w_{n},\theta_{scan})\|]n=1,\ldots ,N_{psh}$$ (6)

Suppose that m sampling angles belong to the scan range. The optimization model can be described as follows:

$$\begin{array}{c} \underset{n_{1},\ldots ,n_{N_{psh}}}{\text{min}}=max_{j=1,\ldots ,m}[\begin{array}{c} \left\|\begin{array}{c} AF(d_{n},w_{n},\theta_{scan}) \end{array}\right\| \end{array}] \end{array}$$ (7)

Where *n*_*min*_ and *n*_*min*_ denote the lower and upper bounds of the phase shifters, and the given upper and lower mask implements the algorithm’s efficiency with minimum mean square error (LMSE) between the desired and the synthesized pattern according to the cost function (C.F). The C.F used here is given by Eq 8;

$$\begin{array}{c} Score=\text{min}[\begin{array}{c} \frac{1}{p}\sum_{i=1}^{p}AF\left(i\right)-Mask\left(i\right) \end{array}] \end{array}$$ (8)

Defining an upper and lower mask should be used to determine the desired pattern. Also, *P* as the number of pattern points should be sufficiently large enough to cover the desired pattern variations. Each pattern’s point that lies outside the specified limits for a given desired radiation pattern contributes value to the cost function.

Distance tapering, amplitude, and phase tapering using the GAPSO hybrid algorithm and controlling the complexity of the feeding network using uniform and non-uniform element grouping simultaneously have caused to get acceptable results from the proposed algorithm.

Other features of this method consist of:

- Solving the problem with the minimum number of phase shifters
- The length of the array antenna is constant
- A fair trade-off between SLL and scan angle by omitting the grating lobe
- The obtained amplitude and phase coefficient being practical, and the ratio of the largest to the smallest of them, respectively being ten and ninety;
- The complexity of the feeding network is being controlled by limiting the repeated usage of subarray elements

Finally, by utilization of a general algorithm to determine the most acceptable design is reported by

- the type of topology of the array
- distance between elements
- complex weight (in other words, means phase shifters and amplifiers)

### 0.2 Design aperiodic array as a BS 5G antenna

A massive antenna with a high gain in the region of 28dB to 35dB is necessary due to the high losses in the mm-wave. To achieve the desired gain could be employed the planner antenna with 256 elements (16*16) up to 2401(49*49) according to the relation between gain and the antenna’s dimension. We acknowledge that the design will get more complicated as the number of elements increases. Afterward, we selected a 49*49 element planner array antenna with 28dB gain. In this paper, to verify the algorithm, the linear array with 49 elements antennas with λ/2 spacing (with the long array of 24.5λ) is designed to synthesize a radiation pattern. The other specifications consist of the value of SLL less than 24 dB, and the frequency center is 28 GHz. The proposed antenna needs to be guaranteed an uncomplicated feeding network; the higher the scan angle, the directive beam, and the more coverage in space with the phase shifter’s minimum number.

**Fig 3:**
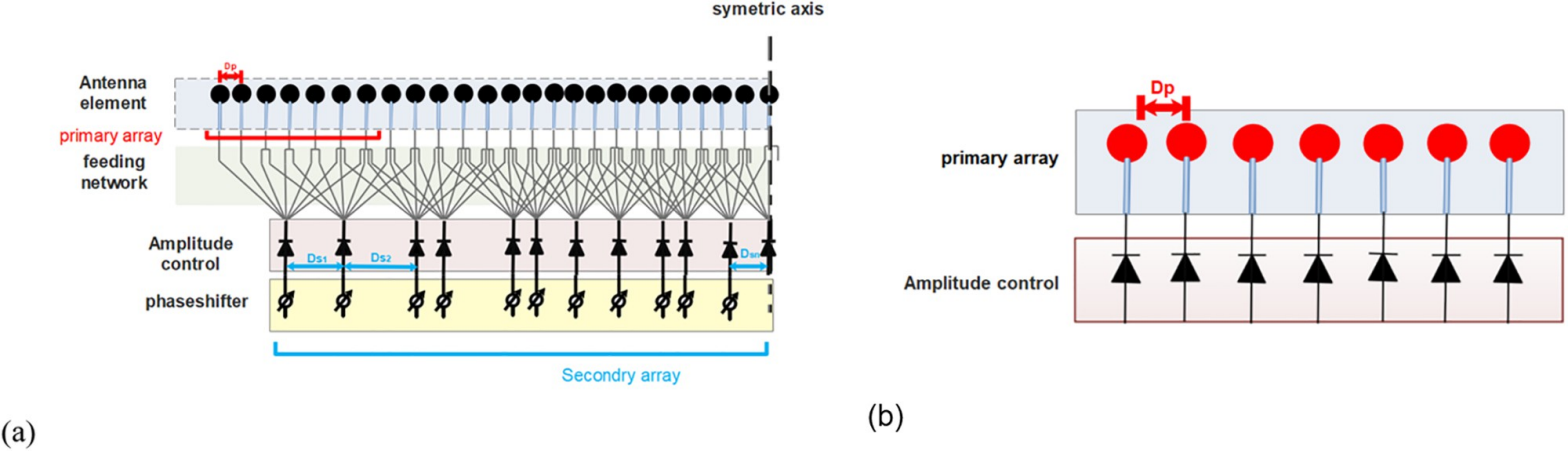
(a) Symmetrical randomly overlap subarray with beam forming unit and amplitude and phase control units (*N*_*total*_ = 49, *N*_*psh*_ = 23, *N*_*p*_ = 7) (b) Uniform primary array groups with equal spacing *D*_*p*_ = λ/2 and symmetrical amplitude control.

In our algorithm, the phase shifters’ number is swept from 7 to 23 to find a better topology from the USA (need 7 phase shifters) as a reference subarray to ROSA (with 23 phase shifters). A linear array diagram composed of 49 elements resulting from our numerical method is given in Fig 3a. As shown in Fig 3a, using one element more than four times is limited to have a more straightforward design. In this simulation, the primary array groups are uniform with equal spacing *D*_*p*_ (Fig 3b) that have been exited only through amplitude.

**Fig 4:**
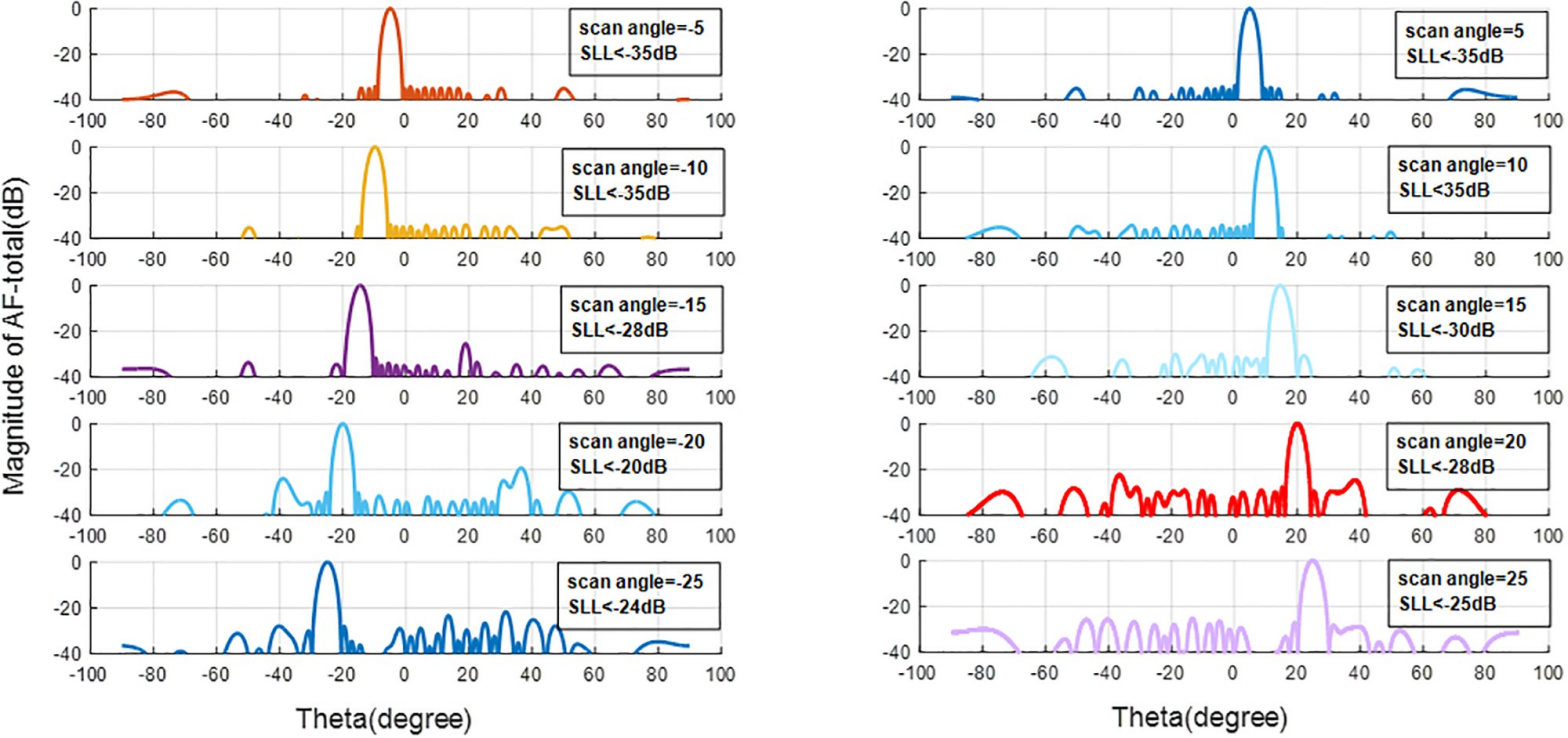
Normalized radiation patterns with GAPSO amplitude and phase tapering for ± 25° scan for aperiodic array in 28 GHz.

The final optimized results are displayed in Fig 4. The desired scan range is ± 25° with SLL better than 24 dB, suitable for the 5G base station antenna With a minimum number of phase shifters. To cover all space, the number of these arrays can use in cylindrical topology with the conformal design of the antenna.

**Fig 5:**
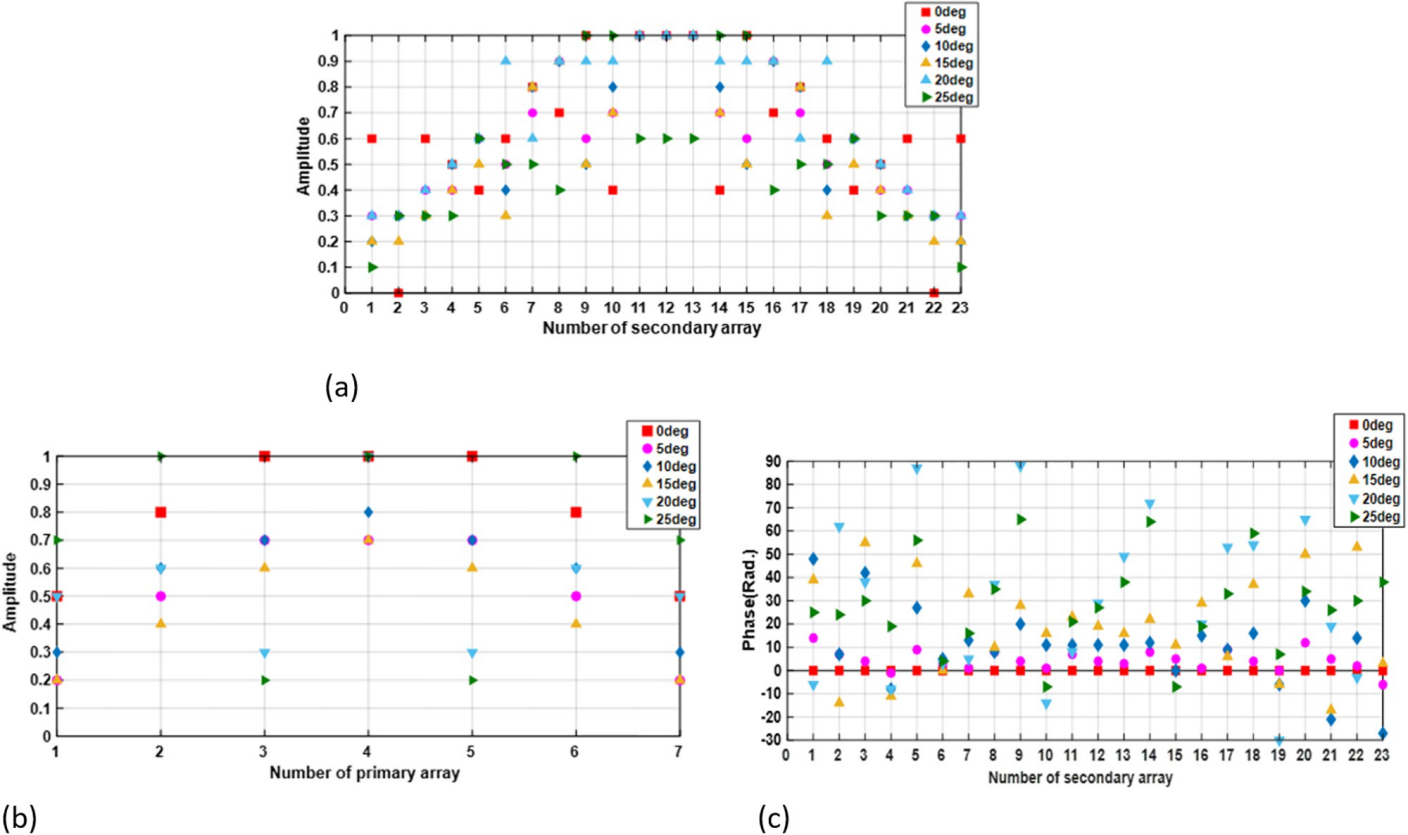
(a) Amplitude of 7 elements in the primary array (b) Amplitude of 23 elements in secondary array (c) phased of 23 elements in secondary array, for scan angle (0°, 5°, 10°, 15°, 20°, 25°).

Fig 5a aillustrates the amplitude of the primary array in different scan angles. The amplitude and the phase of the secondary elements are shown in Fig 5b and 5c.

**Fig 6:**
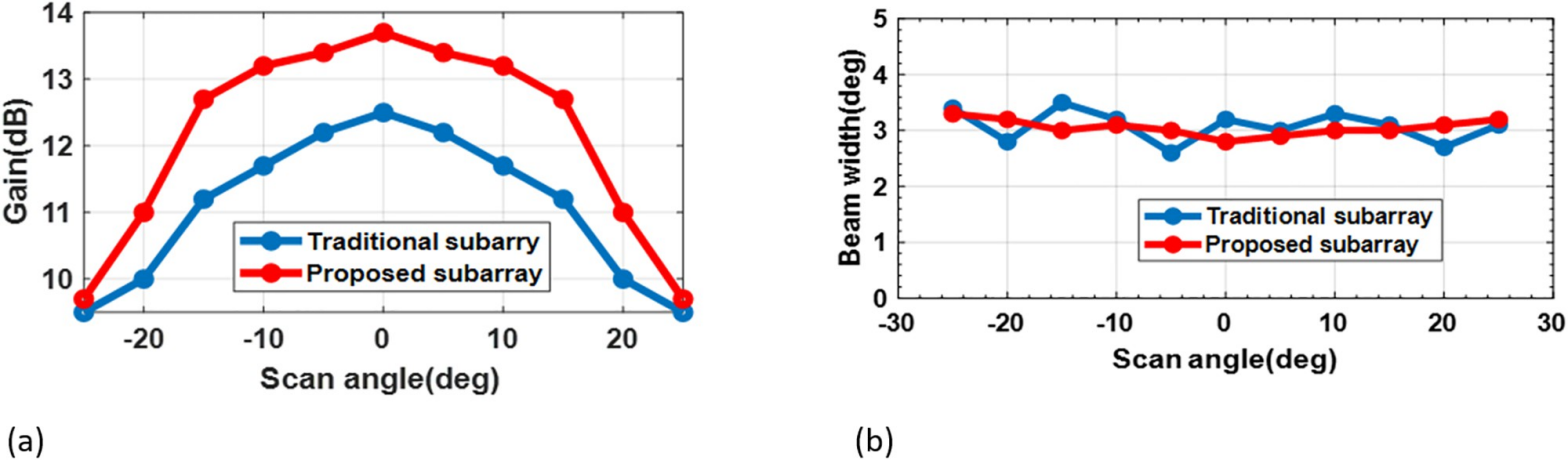
(a) Compare HPBW of the proposed subarray architecture (*N*_*psh*_ = 23) with the filled array architectures. (*N*_*psh*_ = 49). (b) variation of the Gain according to the scan angle for the proposed subarray architecture (*N*_*psh*_ = 23) with the traditional subarray (*N*_*psh*_ = 7).

The results show that instead of 49 phased shifters, we have used 23 to mean our code can diminish the number of phase shifters by 53 percent. The performance of the proposed algorithm is in removing the location of GL compared to the USA where there are six GL in the USA, the distance between the elements is 3.5λ, whereas there is no GL in ROSA. Compared with the recent literature, an achievable improvement in the result is observed. As mentioned, gain and HPBW are changed during scanning in the USA. This variation also exists in our design, but it has been improved. As like, shown in Fig 6a, the variation HPBW is similar to the USA. Still, less than it does, and Fig 6b shows increasing the exact value of the gain and decreasing gain variation of the proposed subarray in comparison with the USA as a traditional subarray.

## Design randomly overlap feeding network

The suggested algorithm can determine the array’s topology to provide the desired scanning angle with specified SLL and the minimum number of phase shifters based on spacing, amplitude, and phase tapering of radiation elements in a large number of phased arrays. For example, 23 phase shifters can obtain the requisite electromagnetic characteristics of the 47-element array (reducing the number of phase shifters by more than 50 percent).

**Fig 7:**
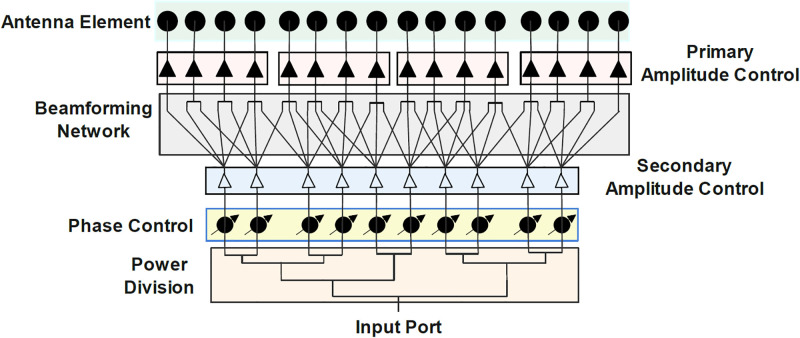
Schematic of the suggested ROSA network for 16-element linear array consisting of power division, phase control, amplitude control of primary and secondary array.

Now, to clarify the feeding network and radiation elements implementation process, a 16-element array with a 4-element subarray to accomplish a gain of 18dB, HPBW of 6.5, SLL of 15dB, and a scanning angle of up to 50° without grating lobe is chosen. The proposed algorithm recommends the ROSA topology with 10 phase shifters to obtain acceptable electromagnetic properties for the selected array, illustrated in Fig 7.

It is important to note that reducing the number of phase shifters from 16 to 10 is not a good criterion for evaluating the proposed algorithm. Since the algorithm is inherently developed for arrays with a large number of elements, the 16-element array is merely presented to illustrate the implementation of the algorithm with an uncomplicated feeding network.

**Fig 8:**
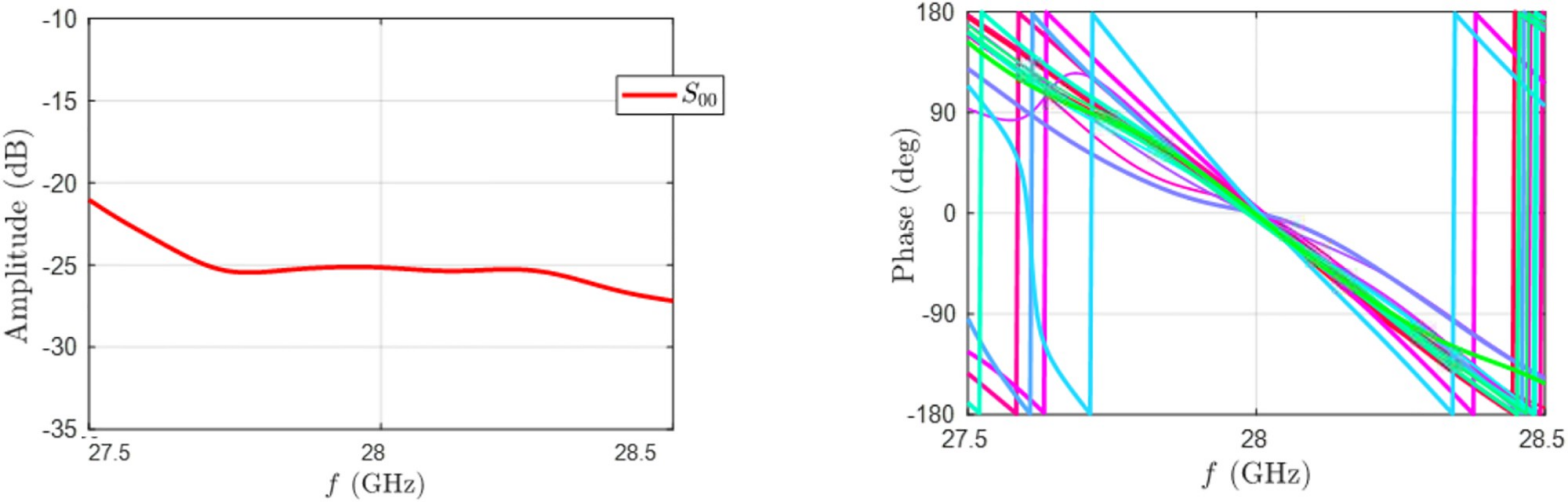
(a) The scattering parameter’s amplitude is less than -22dB, indicating the impedance matching (b) The comparison of the phase profiles of the 16 excitation ports with respect to the input port.

The general structure of a beam-scanning linear array antenna is composed of the following units. A 1-to-10 power divider unit with cascaded unequal T-junction power dividers is used to divide the input power. Next, ten matched adjustable attenuators are used to provide amplitude tapering. The attenuator unit’s output is coupled to the ten adjustable phase-shifters unit, which sets the desired phase shifting to each element to steer the beam. The phase shifter unit’s outputs are fed into the 10-to-16 random overlap beamforming network (BFN). The BFN is a multiple input multiple output configuration consisting of mm-wave passive components such as a T-junction power divider, Wilkinson power combiner, cross-over, and transmission line. Finally, the antenna unit is connected to the output of the random feeding network. The BFN can be described with the scattering matrix (transmission coefficients from the input port to the output port) achieved from full-wave simulation by considering mutual coupling between transmission lines. Fig 8 shows the phase profile and return loss of a BFN, which demonstrates the quality of the suggested design.

**Fig 9:**
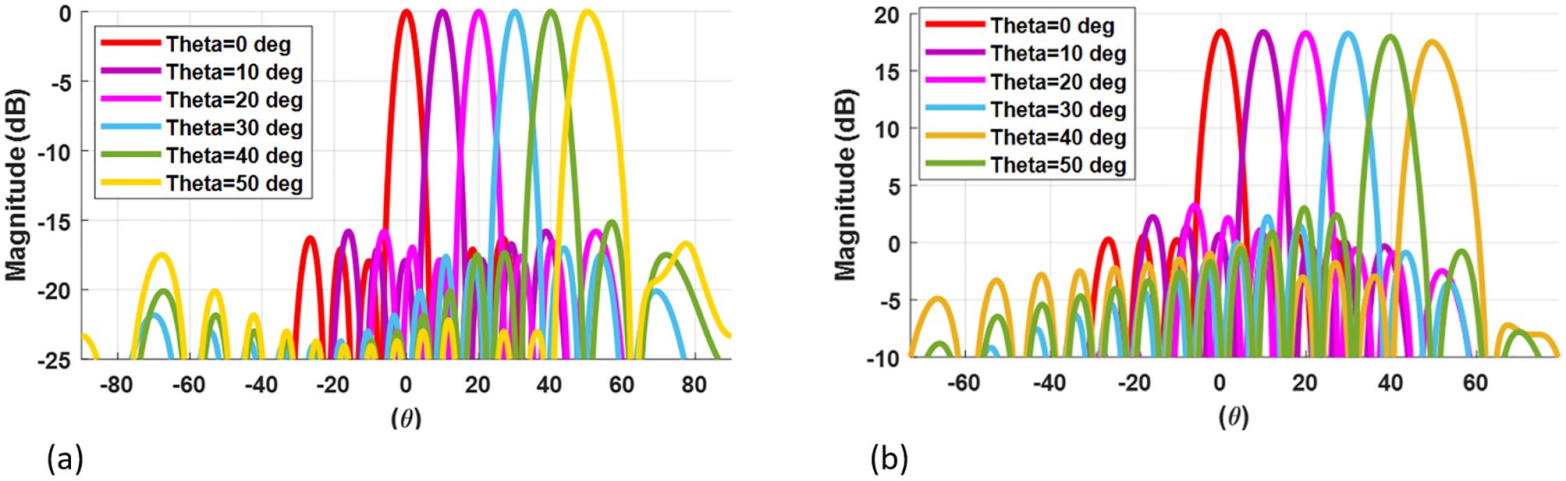
The simulation result of ROSA at 28GHz in a 50° scan sector with a 10° resolution demonstrates that SLL is less than -15dB (a) The array factor from numerical simulation (b) The radiation pattern from full-wave simulation.

The array factor is determined from the excitation coefficients of primary and secondary arrays acquired from the GAPSO algorithm and the scattering matrix of the BFN. The normalized array factor for 50 scanning angle is shown in Fig 9a. The reliability of the ROSA design is evaluated using the CST Full-wave solver, which accounts for mutual coupling between all single elements in 28 GHz. A radiator element is a microstrip patch antenna designed on a Rogers RT/duroid 5880 (*ϵ*_*r*_ = 2.2) matched over the bandwidth and has a maximum gain of 7.9 dBi within the scanning sector.

**Fig 10:**
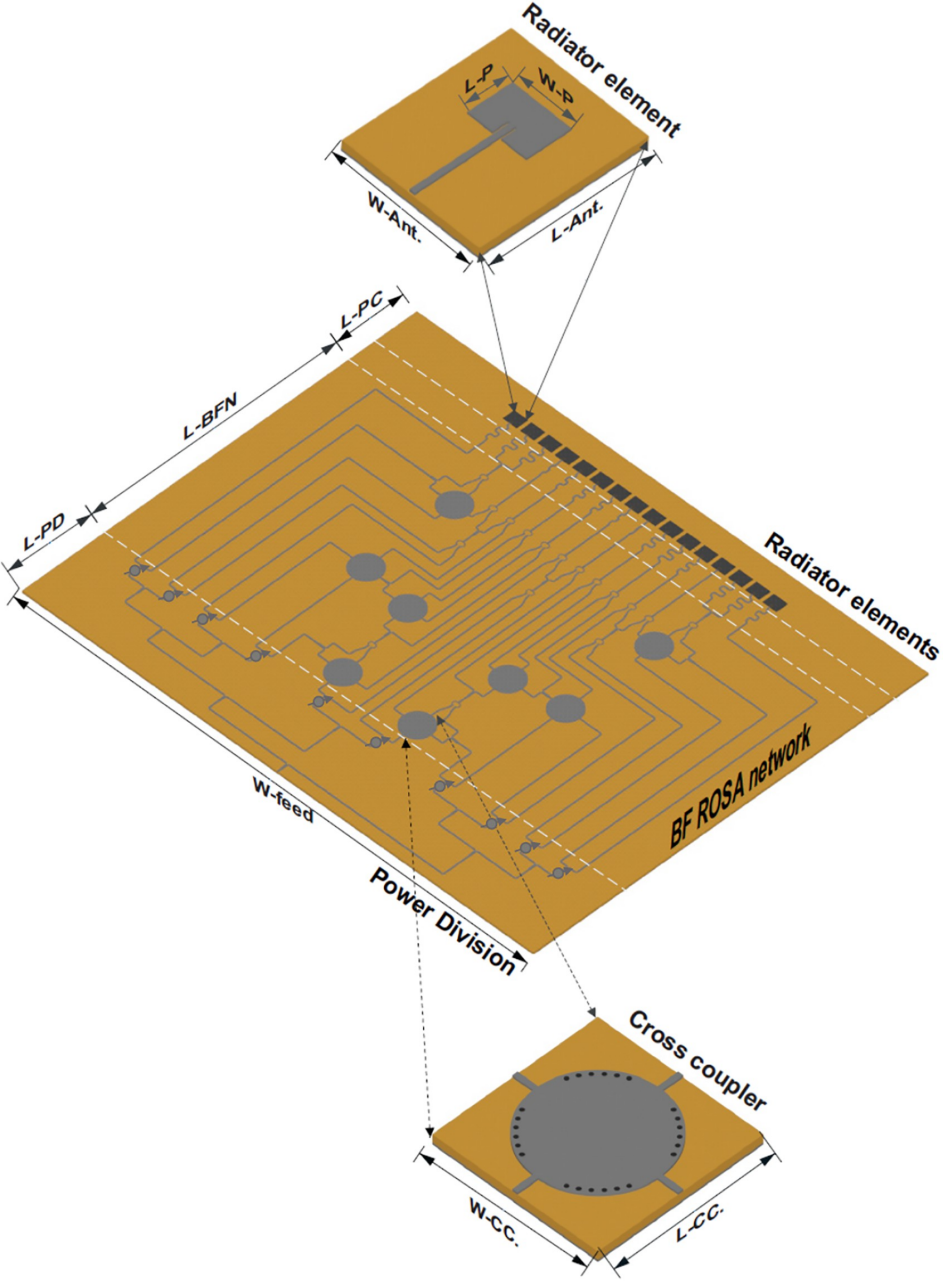
Schematic of the designed ROSA network, including power division, BFN, pase compensation and phase shifter units, for which the geometry parameters are L-PD = 17.54, L-BFN = 84.5, L-PC = 7.75, L-Ant. = 7.5, L-CC. = 8.9, W-feed = 2.3, L-p = 4.3, W-Ant. = 7.5, W-Track = 0.39, W-CC. = 8.9, W-P = 3.9, and tsub = 0.127 all in the unit of mm.

The ROSA feeding network and antenna element are shown in Fig 10 and the different dimensions of devices are on the caption. Even though each element can be excited with three or four input ports, the feeding network has a single-layer structure due to a cross-over element.

The full-wave simulation was used to specify the 16-element radiation pattern, which took mutual coupling between antenna elements into account for a 50° scanning angle with a 10° resolution. The simulation result in Fig 9b. demonstrates that the SLL is better than -15 dB, the gain variation is only 0.8 dB, and the HPBW is less than 1.4° throughout the entire scanning angle.

More specifically, the ROSA provides an SLL that does not exceed -15dB over a scanning angle of up to 50° off-Broadside with a minimum number of phase shifters, as illustrated in Fig 9b.

## Comparing the proposed method with the previous work

Our article’s technique yielded better results by surveying various cases from the literature review and comparing the products and the performance demonstration. Table 2 shows the comparison result in the value of SLL, the phase shifter’s number, the variation of the gain, and HPBW, and the scan angle for the array with *N*_1_-element. In the first case, although the array type is the same in both methods and the number of the phase shifter is used, SLL and the variation of the gain are lower in our algorithm during 8° scanning angles.

**Table 2:** Comparison of the results of our code in with references for some cases.

Gain Variation (*dB*)	*f*(*GHz*) *Ref*.	*N*_1_	Tape array	*N*_*psh*_	scan angle	*SLL*(*dB*)	HPBW Variation (*deg*)
2*1 0.9	2*10 [17]	2*50	ROSA	16	± 8°	-16	-
0.7	our code		ROSA	16	± 8°	-28	2.4
2*2 2	2*7.9 [18]	2*32	RSA	13	± 20°	-15	-
1.8	our code		ROSA	12	± 20°	-26	1.4
2*3 -	2*30 [18]	2*30	RSA	12	± 14°	-15	-
2.1	our code		ROSA	14	± 21°	-24	2.7

It is because of using a better algorithm to determine complex weighting, choosing the best topology for the array by considering all ROSA combinations according to the cost function. Also, in case 2, our code’s effectiveness shows the smaller number of phase shifters, the better the result. For the third case, using two additional phase shifters significantly affects the design proposed by our algorithm. Also, the HPBW changes observed in this paper and Other references did not report the HPBW variations.

**Fig 11:**
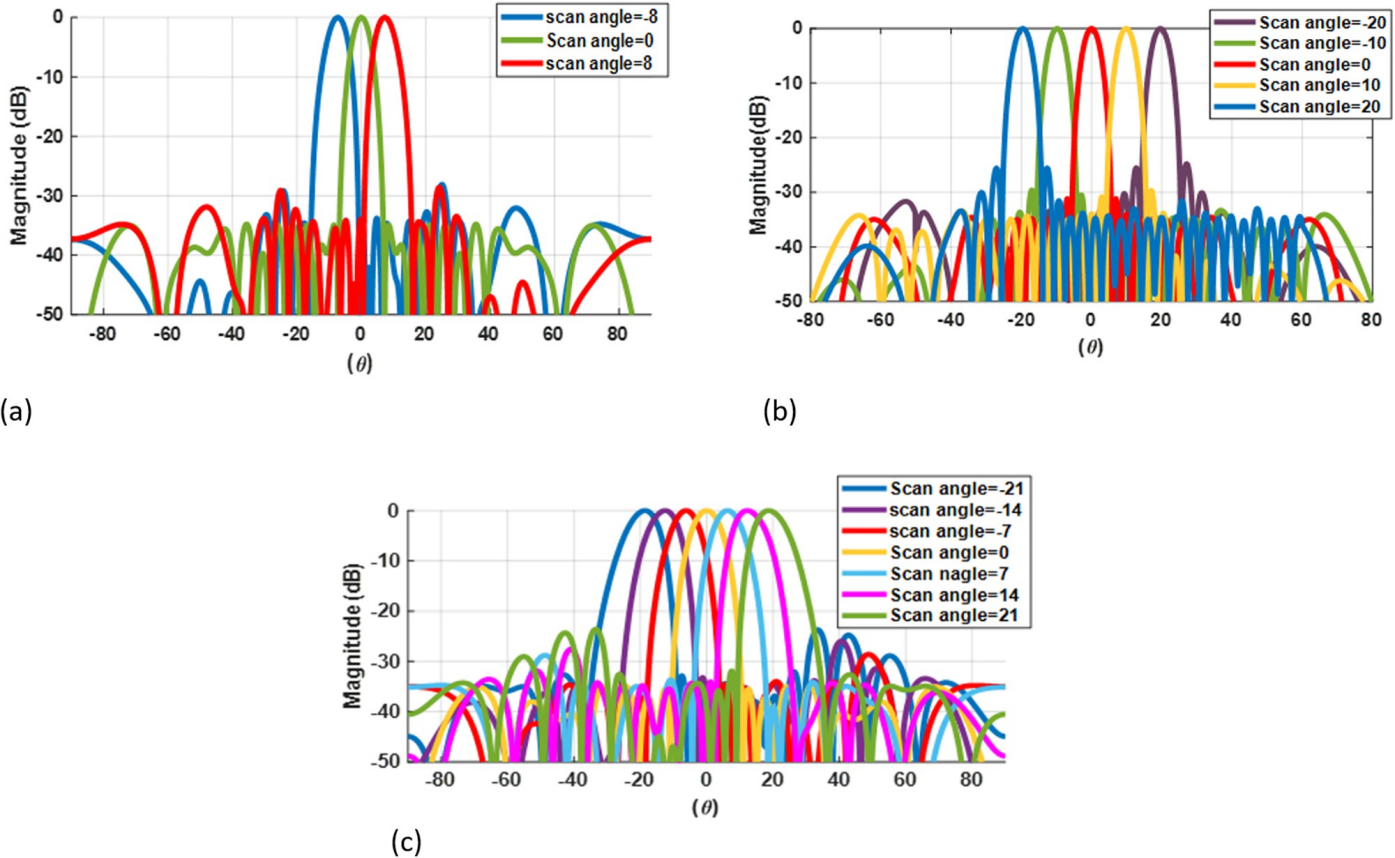
Total array factor (product of primary and secondary array) for three cases: (a) linear array with 50-elements in 10GHz and ± 8°scan angle (b) array with 32 elements that scan from -20° to +20° (c) 30-element array with 21° as a scan angle.

The radiation pattern of the above examples is shown in Fig 11. As shown in the figure, the result of three cases by our algorithm can respond better in SLL and give a more comprehensive scan angle. Because our algorithm determines the best topology among all possible plans from the USA to UOSA, ROSA, and ROA.

## Conclusion

This paper proposes the general algorithm as a practical toolbox for designing an affordable phased array antenna according to the variation of spacing elements and complex coefficients tapering. In the suggested methodology, the placement of the antenna element is determined numerically based on the number of primary and secondary arrays to achieve a wide scan angle without a grating lobe. Afterward, the GAPSO optimization method was applied to obtain implementable excitation coefficients for managing SLL in region scan angle. A straightforward feeding network is a prerequisite for solving the problem. It is used to design a 5G base station antenna with 47-element to gain 28 dB in 28 GHz, demonstrating our method’s applicability. This antenna has a low cost and a simple setup; the accomplished result includes a 53 percent reduction in phase shifters, the removal of GL, and a maximum scan angle of *pm* 25*circ* with 24 dB as SLL. Additionally, to clarify how to implement the aperiodic arrays’ feeding network, a 16-element array is designed in a full-wave simulation that considers the effect of the mutual coupling of elements.
